# Environmental Factors Contributing to the Spread of H5N1 Avian Influenza in Mainland China

**DOI:** 10.1371/journal.pone.0002268

**Published:** 2008-05-28

**Authors:** Li-Qun Fang, Sake J. de Vlas, Song Liang, Caspar W. N. Looman, Peng Gong, Bing Xu, Lei Yan, Hong Yang, Jan Hendrik Richardus, Wu-Chun Cao

**Affiliations:** 1 Beijing Institute of Microbiology and Epidemiology, State Key Laboratory of Pathogen and Biosecurity, Beijing, People's Republic of China; 2 Department of Public Health, Erasmus Medical Center (MC), University Medical Center Rotterdam, Rotterdam, The Netherlands; 3 College of Public Health, The Ohio State University, Columbus, Ohio, United States of America; 4 State Key Laboratory of Remote Sensing Science, Chinese Academy of Sciences, Beijing Normal University, Beijing, People's Republic of China; 5 Department of Environmental Science, Policy and Management, University of California, Berkeley, United States of America; 6 Department of Geography, University of Utah, Salt Lake City, Utah, United States of America; U.S. Naval Medical Research Center Detachment/Centers for Disease Control, United States of America

## Abstract

**Background:**

Since late 2003, highly pathogenic avian influenza (HPAI) outbreaks caused by infection with H5N1 virus has led to the deaths of millions of poultry and more than 10 thousands of wild birds, and as of 18-March 2008, at least 373 laboratory-confirmed human infections with 236 fatalities, have occurred. The unrestrained worldwide spread of this disease has caused great anxiety about the potential of another global pandemic. However, the effect of environmental factors influencing the spread of HPAI H5N1 virus is unclear.

**Methodology/Principal Findings:**

A database including incident dates and locations was developed for 128 confirmed HPAI H5N1 outbreaks in poultry and wild birds, as well as 21 human cases in mainland China during 2004–2006. These data, together with information on wild bird migration, poultry densities, and environmental variables (water bodies, wetlands, transportation routes, main cities, precipitation and elevation), were integrated into a Geographical Information System (GIS). A case-control design was used to identify the environmental factors associated with the incidence of the disease. Multivariate logistic regression analysis indicated that minimal distance to the nearest national highway, annual precipitation and the interaction between minimal distance to the nearest lake and wetland, were important predictive environmental variables for the risk of HPAI. A risk map was constructed based on these factors.

**Conclusions/Significance:**

Our study indicates that environmental factors contribute to the spread of the disease. The risk map can be used to target countermeasures to stop further spread of the HPAI H5N1 at its source.

## Introduction

The H5N1 subtype of the influenza A virus was initially detected in poultry on a farm of Scotland, UK, in 1959 [1]. The highly pathogenic avian influenza (HPAI) virus reappeared in 1997 and caused an outbreak in chicken farms and live bird markets in Hong Kong, where 18 human cases were reported with 6 deaths [2]. The recent chain of outbreaks caused by H5N1 started among poultry in South Korea in December 2003, and has affected 61 countries in Asia, the Middle East, Africa and Europe leading to the deaths of millions of poultry and more than 10 thousands of wild birds [3], [4]. Even worse, the HPAI H5N1 virus appears to have gained ability to cross the species barrier and induce severe disease and death in humans as well as other mammals.

As of 18-March 2008, there have been 373 laboratory-confirmed human infections, of which 236 have died [5]. The worldwide spread of the disease is providing more opportunities for viral re-assortment within a host (genetic shift) and mutation over time (genetic drift). These factors may lead to a viral strain that is more efficient at person-to-person transmission, raising the potential for another pandemic to occur [6]–[9]. Since January 2004, HPAI H5N1 outbreaks in poultry and wild birds, and occasional trans-species transmission to humans have been reported throughout mainland China [5], [10]. Surveillance studies suggested that poultry movement and wild bird migration may have contributed to such a quick spread [7], [11], [12]. However, the processes, including environmental factors, influencing the spread of HPAI H5N1 virus are not clearly understood.

In this study, we explore environmental factors associated with such outbreaks in mainland China to provide essential information for developing effective and appropriate countermeasures.

## Results

**Figure 1:**
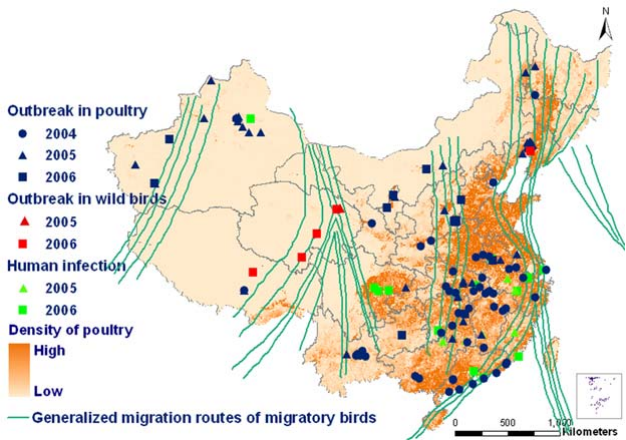
Spatial distribution of HPAI H5N1 outbreaks in domestic poultry and wild birds and human cases in mainland China. Poultry density is indicated by color gradient. Distributions of generalized migration routes of migratory birds are overlapped.

Since the emergence of HPAI H5N1 infections in mainland China in January 2004, a total of 128 outbreaks of HPAI H5N1, spanning a large geographic area of mainland China, has occurred in poultry and wild birds at the village/township level in 26 of 31 provinces, municipalities or autonomous regions by the end of 2006 [10]. The spatial distributions of HPAI H5N1 outbreaks in poultry and wild birds, and human cases in mainland China were displayed in the thematic map (Figure 1). The background of the map was the poultry density. The generalized migration routes of birds were overlapped on the map.

In a case-control study, minimal distances to the nearest lake, wetland, national highway and main city, as well as annual precipitation appeared to be significantly associated factors in the univariate analysis (Table 1). Multivariate logistic regression demonstrated that three variables, minimal distance to the nearest national highway, annual precipitation and the interaction between minimal distance to the nearest lake and wetland, were significantly associated with HPAI H5N1 outbreaks (Table 1). Goodness of fit for the logistic regression model was evaluated using Hosmer-Lemeshow test, showing a high risk discrimination between outbreak sites and “control” areas (X^2^ = 4.305, *P* = 0.829).

**Table 1:** The association between H5N1 outbreaks and influential factors by logistic regression analysis.

Influencing factors (Unit)	Univariate analysis		Multivariate analysis	
	Crude OR* (95% CI)	*P*-value	Adjusted OR* (95% CI)	*P*-value
Minimal distance to the nearest body of water (10 km)				
Minimal distance to the nearest lake	0.715 (0.612∼0.835)	<0.001		
Minimal distance to the nearest reservoir	0.996 (0.996∼1.027)	0.800		
Minimal distance to the nearest river	1.029 (0.999∼1.060)	0.062		
Minimal distance to the nearest wetland (10 km)	0.949 (0.918∼0.980)	0.001		
Minimal distance to the nearest transportation routes (10 km)				
Minimal distance to the nearest railway	0.998 (0.983∼1.013)	0.799		
Minimal distance to the nearest freeway	1.002 (0.992∼1.011)	0.693		
Minimal distance to the nearest national highway	0.813 (0.711∼0.929)	0.002	0.842 (0.742∼0.956)	0.008
Minimal distance to the nearest bird migration route (10 km)	1.008 (0.988∼1.029)	0.434		
Minimal distance to the nearest main city (10 km)	0.948 (0.902∼0.997)	0.039		
Climate				
Annual precipitation (100 mm)	0.932 (0.892∼0.973)	0.001	0.913 (0.873∼0.954)	<0.001
Elevation (100 m)	1.004 (0.984∼1.024)	0.696		
Poultry density (1000/km^2^)	0.997 (0.986∼1.007)	0.549		
Interaction between minimal distance to the nearest lake and wetland (10 km * 10 km)			0.969 (0.955∼0.983)	<0.001

*ORs of the variables involving minimal distances to the nearest water bodies, wetlands, transportation routes, bird migration routes and main cities were calculated for a ten-kilometer difference, ORs were estimated regarding annual precipitation for a 100-millimeter difference, elevation for a 100-meter difference and poultry density for 1000-bird difference per square kilometer.

We have also investigated possible overestimation of effects due to clustering of neighboring outbreaks. Within clusters, transmission of H5N1 from one location to another could have occurred directly, instead of through the investigated environmental factors. Using the criterion of a distance <20 km and a time-interval <3 weeks, we could identify 6 clusters in a total of 30 outbreaks. We have repeated the multivariate logistic regression using only one randomly selected outbreak site from each cluster (i.e., 104 cases and 520 controls), but this did not lead to significantly different results. The adjusted OR and *P*-value for the three factors, minimal distance to the nearest national highway, annual precipitation and the interaction between minimal distance to the nearest lake and wetland, were 0.825 (*P* = 0.005), 0.915 (*P* = 0.001), and 0.970 (*P*<0.001) respectively.

**Figure 2:**
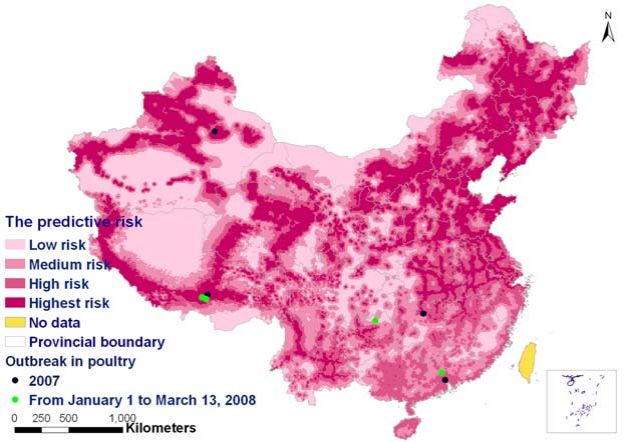
Predictive risk map of HPAI H5N1 outbreaks in birds. The predictive risk map shows increased risk of HPAI H5N1 outbreaks in poultry and wild birds by color gradient. Locations of HPAI H5N1 outbreaks in poultry from January of 2007 to March 13, 2008 are also indicated (8 HPAI H5N1 outbreaks in mainland China).

Based on the predictive model derived from the logistic regression analysis, a predictive risk map of HPAI H5N1 infections was established for mainland China by GIS technologies (Figure 2). On the risk map, the locations of HPAI H5N1 outbreaks in poultry from January of 2007 to March 13, 2008 were also plotted (8 outbreaks). The overlapping analysis revealed that 87.5% (7/8) of outbreaks of HPAI H5N1 in poultry occurred in the predictive highest or high-risk areas (Figure 2).

## Discussion

The results of our case-control study demonstrate that the interaction between minimal distance to the nearest lake and wetland minimal distance to the nearest national highway, and the annual average precipitation are the principal environmental variables contributing to the spread of HPAI H5N1 virus in mainland China. These findings indicate that bird populations in close proximity to a body of water are in danger of becoming infected, and provide further evidence for the role of waterfowl in the transmission of avian influenza. HPAI is mainly characterized by a quick spread and a high mortality rate in poultry. However, except for some species [13], waterfowl are typically asymptomatic reservoirs for H5N1 [14], perhaps shedding virus through their salivary and nasal secretions and feces into bodies of water in which they are inhabiting [11]. It is estimated that the virus can survive in water at 22°C for up to 4 days and up to 30 days at 0°C [15]. People may unconsciously take the virus to a body of water from contaminated surfaces or infected birds. Birds and other animals also may transport the virus on their feathers or fur to a water source after coming into contact with an infected animal or contaminated surface on a farm. The spread of the virus is facilitated when the body of water, such as a lake, is stagnant and adjacent to a farm or community [16]. Interestingly, as the precipitation in a region increases, the risk of HPAI H5N1 outbreaks is reduced. One explanation for this result may be that lower precipitation levels may lead to a higher concentration of birds in a reduced number of wetlands, thus increasing the chances of bird becoming infected through contact with the virus.

Proximity to national highway is another risk factor contributing to HPAI infection. National highways in mainland China are funded and constructed by the central government and are vital connections between provinces, without collection of toll. As there are many restrictions on railway transportation of poultry in mainland China [17], and tolls on freeways are quite high, national highways are usually top-priority to transport poultry and their products throughout the country. During long-distant transportation, a variety of birds and animals from various origins are caged on top of each other, perhaps providing an easy way of cross-infection of avian influenza. In addition, many open live poultry markets are established along or near the national highways, which may further increase the chance of virus transmission. These findings suggest that trade and mechanical movement of poultry may facilitate spread of HPAI H5N1 virus, supporting laboratory evidence as demonstrated by Li et al. [9].

The logistic regression analysis in the study demonstrates that the risk of HPAI H5N1 infections are not increased with the poultry density, as is usually presumed. This may due to the fact that poultry, especially chickens in the areas with high population densities, are usually bred in industrialized farms with good animal husbandry practices and properly vaccinated [18]. Although the importance as an ecological reservoir is uncertain, migratory birds may spread H5N1 viruses to new geographic regions. Usually migratory birds cannot fly the full distance to their annual migratory destination. Instead, they usually interrupt their migration to rest and refuel [19]. Avian influenza may be spread between wild and domestic birds when migratory birds search for food, water and shelter. The infected wild birds can carry the influenza virus for long distances during migration [20]. Migratory birds are loyal to their annual migratory destinations and their stopover points, such as water bodies, wetlands and forests [21]. As wetlands and forests are destroyed however, due to increased human activities, especially land utilization practices, migratory birds may be forced to search for shelter and food in other places such as farms. This may result in increases contact between wild and domestic birds, thus facilitating the transmission of the virus to domestic bird populations.

In conclusion, the analyses of the spatial distribution and underlying environmental determinants reveal that the spread of the HPAI H5N1 is probably taking place at two different but interlinked patterns. Transportation of poultry and their products along highways may contribute to the long-distant national wide spread of the disease. Contacts with infected birds, trade and mechanical movement of poultry may be responsible for local transmission. The two spread patterns can exist simultaneously, and HPAI H5N1 outbreaks can take place near national highways, near relatively stagnant bodies of water such as lakes and wetlands, and in particular when there is reduced rainfall. The predictive risk map of HPAI H5N1 infections established for mainland China on basis of the above contributing factors may be useful for identifying the areas where surveillance, vaccination and other preventive interventions should be targeted.

## Materials and Methods

### Data collection and management

The data on HPAI H5N1 outbreaks in animals were obtained from monthly reports, the official veterinary bulletins of the Ministry of Agriculture of China and from updates on HPAI in animals from the World Organization for Animal Health (OIE) [3], [22]. All the outbreaks were confirmed with laboratory based virological methods and officially reported in mainland China.

We developed a database including the information on the incident dates (rather than the dates of reporting) and locations of outbreak in birds. The information on human cases with H5N1 infection in mainland China was also included in the database. Each of the HPAI H5N1 outbreaks in birds, as well as human infections, were geo-coded at the village/township level and linked to a digital map at the scale of 1∶100,000 using geographical information system (GIS) technologies. Point-type information (single pair coordinates) was created for each outbreak site, while line-type information was generated for migration routes of migratory birds, based on detailed bird banding records from mainland China [23]. Polygon-type information for water bodies were derived from digital maps. The three-type information was overlapped for analyses in our study. Water bodies included lakes with a surface AREA ≥1.0 km^2^, reservoirs having a surface area ≥1.0 km^2^ and both were used as polygon-type map layers. In addition, information on transportation, main cities and elevation were directly obtained from digital maps (provided by the coauther Dr. Peng Gong from State Key Laboratory of Remote Sensing Science). According to the definition suggested by U.S. Fish and Wildlife Service [24], [25], wetlands stated in the current study only included swamps, water meadows, wading lakes with a surface ≥1.0 km^2^, and excluded rivers, reservoirs and deep lakes. The data on wetlands were obtained from the National Geographical Resourse Center, which were derived from wetland census data collected in mainland China. These data were digitized as point-type information.

Transportation including railways, freeways and national highways were used as line-type information. Main cities included 31 provincial capitals and 305 prefecture-level cities in mainland China and were used as point-type information. The layer of elevation was used as line-type information and was preprocessed to convert it to a raster-type layer for this study. The raster-type map layer of precipitation extrapolated by the kriging technique using 700 weather stations in mainland China was collected from the Institute of Geographical Sciences and Natural Resources Research, Chinese Academy of Sciences. Poultry density information was obtained from the FAO [26], which was a raster-type layer and was as the predicted poultry density, corrected for unsuitability and adjusted to match observed totals.

### GIS spatial analysis

The spatial distribution of outbreaks in birds and human cases was studied through overlapping analysis. A thematic map was established on which the poultry density was taken as the background. To understand the role of bird migration in the spread of H5N1 virus, map layer of bird migration was created and overlapped on the map of spatial distribution of outbreaks in birds and human cases.

### Analysis of environmental factors associated with H5N1 outbreaks

**Figure 3:**
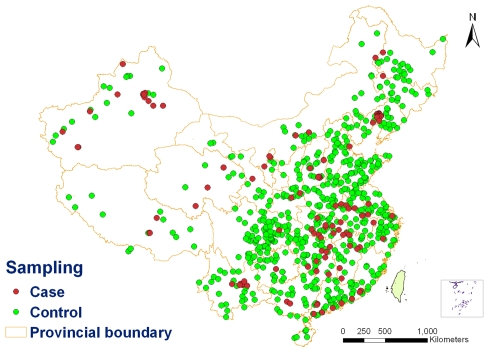
Spatial random sampling for the case-control design. This figure highlights the case and control sites selected by the spatial random sampling approach.

A case-control study design was used to clarify the environmental factors associated with the spread of HPAI H5N1. The 128 outbreak sites were taken as “cases”. All other villages and townships in mainland China except for those affected by HPAI H5N1 outbreaks from 2004 to 2006, were defined as non-epidemic areas. Then 640 “control” sites (5 controls/case) were randomly selected from the non-epizootic areas in mainland China and then were geo-coded (see Figure 3 for the location of cases and controls). Eight environmental factors (bodies of water, wetlands, transportation routes, migration routes, main cities, precipitation, elevation and poultry density) involving twelve variables were considered in the study. The minimum distances to the nearest bodies of water including lakes, reservoirs and rivers, as the polygon-type information, were measured using a proximity function of spatial analysis in such an algorithm that the minimal distance from each case and control site to its nearest water body edge was calculated. The minimal distance from each “case/control” site to its nearest points or lines was calculated, using point-type or line-type information (i.e., wetlands, transportation routes including railways, freeways and national highways, migration routes and main cities). Furthermore, using a zonal statistical calculation technique, an 8 km mean buffer zone (the outbreak area of 3 km plus risk area of 5 km around the outbreak site) was calculated for the variables, annual precipitation, elevation and poultry density (as raster-type map layers), for each case and control site.

Statistical analyses were performed using the Statistical Package for Social Sciences (SPSS Inc, Chicago, IL, USA). Unconditional logistic regression was performed, and odds ratios (ORs), their 95% confidence intervals (CIs) and *P-*values were estimated using maximum likelihood methods. ORs of the variables involving minimal distances to the nearest body of water, wetlands, transportation routes, bird migration routes and main cities were calculated for a ten-kilometer difference. However, for annual precipitation for a 100-millimeter difference, elevation for a 100-meter difference and poultry density for 1000-bird difference per square kilometer, ORs were estimated respectively. Univariate analyses were conducted to examine the effect of each variable separately. Quadratic and logarithmic transformations of each variable were also tested, but these did not perform significantly better than a linear association for any of the variables. Multivariate analysis was then performed using the variables with *P*-value of <0.1 from the univariate analyses as covariates. The possible interactions between each covariate were also included in the multivariate analysis. The colinearity between covariate in the case control study was quantitatively assessed. Correlations between minimal distances to the nearest lake, minimal distance to the nearest wetland and minimal distance to the nearest river were identified. Models were also optimized by comparing the −2 log likelihood and Hosmer-Lemeshow goodness of fit when correlated variables were added or removed. It was discovered that more accurate models could be derived by removing the variable of minimal distance to the nearest river. A *P*-value <0.05 was considered statistically significant by using backward-LR method. Goodness of fit for the logistic regression model was evaluated by using the Hosmer-Lemeshow goodness of fit test.

To predict the risks of HPAI H5N1 occurrence, a grid map was created using GIS techniques. The values of the above predictive variables were determined for each grid with an area of 100 km^2^ (10×10 km) based on the predictive model derived from the multivariate logistic regression analysis. By interlinking all the grids, a predictive risk map of HPAI H5N1 infections was established for mainland China. The risk of HPAI occurrence for each grid was calculated and classified as the highest risk, high risk, medium risk and low risk according to quartile levels for predicted prevalence in the predictive map.
